# Duplication of the IL2RA locus causes excessive IL-2 signaling and may predispose to very early onset colitis

**DOI:** 10.1038/s41385-021-00423-5

**Published:** 2021-07-05

**Authors:** Maria E. Joosse, Fabienne Charbit-Henrion, Remy Boisgard, Rolien (H.) C. Raatgeep, Dicky J. Lindenbergh-Kortleve, Léa M. M. Costes, Sandrine Nugteren, Nicolas Guegan, Marianna Parlato, Sharon Veenbergen, Valérie Malan, Jan K. Nowak, Iris H. I. M. Hollink, M. Luisa Mearin, Johanna C. Escher, Nadine Cerf-Bensussan, Janneke N. Samsom

**Affiliations:** 1 Laboratory of Pediatrics, division Gastroenterology and Nutrition, Erasmus University Medical Center-Sophia Children’s Hospital, Rotterdam, the Netherlands; 2 Laboratory of Intestinal Immunity, Université de Paris, Imagine Institute, INSERM UMR 1163, Paris, France; 3 Department of Molecular Genetics, Université de Paris, Necker-Enfants Malades Hospital, Paris, France; 4 GENIUS group from the European Society for Paediatric Gastroenterology, Hepatology and Nutrition (ESPGHAN), http://www.genius-group.org; 5 Department of Cytogenetics, Université de Paris, Necker-Enfants Malades Hospital, Paris, France; 6 Department of Pediatric Gastroenterology and Metabolic Diseases, Poznan University of Medical Sciences, Poznan, Poland; 7 Department of Clinical Genetics Erasmus University Medical Center, Rotterdam, the Netherlands; 8 Department of Pediatrics, Unit of Pediatric Gastroenterology, Leiden University Medical Center, Leiden, the Netherlands; 9 Department of Pediatric Gastroenterology, Erasmus University Medical Center-Sophia Children’s Hospital, Rotterdam, the Netherlands

## Abstract

Single genetic mutations predispose to very early onset inflammatory bowel disease (VEO-IBD). Here, we identify a de novo duplication of the 10p15.1 chromosomal region, including the *IL2RA* locus, in a 2-year-old girl with treatment-resistant pancolitis that was brought into remission by colectomy. Strikingly, after colectomy while the patient was in clinical remission and without medication, the peripheral blood CD4:CD8 ratio was constitutively high and CD25 expression was increased on circulating effector memory, Foxp3^+^, and Foxp3^neg^ CD4^+^ T cells compared to healthy controls. This high CD25 expression increased IL-2 signaling, potentiating CD4^+^ T-cell-derived IFNγ secretion after T-cell receptor (TCR) stimulation. Restoring CD25 expression using the JAK1/3-inhibitor tofacitinib controlled TCR-induced IFNγ secretion in vitro. As diseased colonic tissue, but not the unaffected duodenum, contained mainly CD4^+^ T cells with a prominent IFNγ-signature, we hypothesize that local microbial stimulation may have initiated colonic disease. Overall, we identify that duplication of the *IL2RA* locus can associate with VEO-IBD and suggest that increased IL-2 signaling predisposes to colonic intestinal inflammation.

## Introduction

Inflammatory bowel disease (IBD) results from aberrant immune responses to intestinal microbiota and is maintained by inflammatory CD4^+^ effector T cells that have specificity for microbial antigens and reside in the intestinal lamina propria.^1,2^ There is a large variability in clinical disease patterns and, despite a growing availability of new therapeutic options, 40–50% of the patients suffer from frequent relapses or continuous inflammation. Identification of the patient’s underlying immune disease and subsequent tailoring of treatment is therefore highly desired. Although genome-wide association studies have suggested that genetic control of inflammatory T-cell responses is linked to IBD susceptibility,^3^ the functional impact of most key susceptibility genes associated with IBD is currently not understood.^4,5^ Rare single genetic mutations can predispose to very early onset inflammatory bowel disease (VEO-IBD), an IBD-like disease presenting before the age of six. Monogenic defects, such as the *IL10, IL10R,* and *FOXP3* loss-of-function mutations causing VEO-IBD, have uncovered pathways that are essential to prevent intestinal inflammation.^6,7^ As these monogenic defects fall within inflammatory immune networks that overlap with polygenic IBD loci, in depth immunological characterization of VEO-IBD patients provides key information to advance IBD patient classification.

One of the key genes shared between IBD susceptibility loci and monogenic VEO-IBD is the *IL2RA* gene encoding the interleukin-2 receptor alpha chain (CD25).^8^ CD25 is constitutively expressed by CD4^+^Foxp3^+^ regulatory T cells, on a subset of circulating CD4^+^ memory T cells and is rapidly induced on effector CD4^+^ T cells after T-cell receptor signaling.^9,10^ CD25 is the low-affinity IL-2 receptor which cannot function independently but forms a high-affinity IL-2 receptor when associated with the IL-2Rβ and common γ chain.^11^ Signaling through the IL-2R induces T-cell proliferation and is critical for the development and peripheral expansion of CD4^+^CD25^+^ regulatory T cells.^12,13^ As a result, IL-2 signaling is essential for intestinal homeostasis and both *Il2ra−/−* and *Il2−/−* mice develop spontaneous colitis, the latter with a predominant CD4^+^ T cell infiltration in the lamina propria.^14–16^ In analogy, the clinical disease in *IL2RA* deficient patients resembles deficiency in the *FOXP3* gene causing polyendocrinopathy, enteropathy, X-linked (IPEX) syndrome-like disease.^8^ IL-2 shortage has been associated with autoimmune inflammation in multiple diseases such as systemic lupus erythematosus (SLE) and diabetes.^17,18^ Clinical trials with low-dose human-recombinant IL-2 supplementation are ongoing and show potent immunosuppressive effects in chronic graft-versus-host disease.^19^ Paradoxically, an early study showed that high dose IL-2 treatment in cancer causes gastrointestinal side effects in the majority of patients, including nausea, vomiting and diarrhea.^20^ Moreover, excessive CD25 expression also occurs in several autoimmune diseases and its inhibition effectively blocks clinical and inflammatory disease activity.^21,22^ Together, these data argue that a balanced IL-2 regulation is pivotal for intestinal homeostasis. However, it is unknown how intrinsically high IL-2 signaling would affect intestinal immune responses and to what degree it is detrimental for intestinal homeostasis.

Here, we identify a de novo duplication of the 10p15.1 chromosomal region, including the *IL2RA* gene, in a 2-year-old female patient presenting with therapy-resistant VEO-IBD that was brought into remission by subtotal colectomy. Our data demonstrate that the patient’s CD4^+^ T cells exhibit constitutive activation of the IL-2R-pSTAT5 pathway leading to hyper-responsiveness of CD4^+^ effector T cells possibly predisposing to T-cell driven pancolitis. As diseased colonic tissue, but not the unaffected duodenum, contained mainly CD4^+^ T cells with a prominent IFNγ signature, we hypothesize that local microbial stimulation may have initiated colonic disease. These findings shed new light on the role of IL-2 in intestinal homeostasis and direct further studies to examine the functional consequences of *IL2RA* genetic variation in IBD patients.

## Results

### In a case of VEO colitis, CGH identifies a de novo 374 kb duplication of the 10p15.1 region containing the *IL2RA* locus

**Fig. 1:**
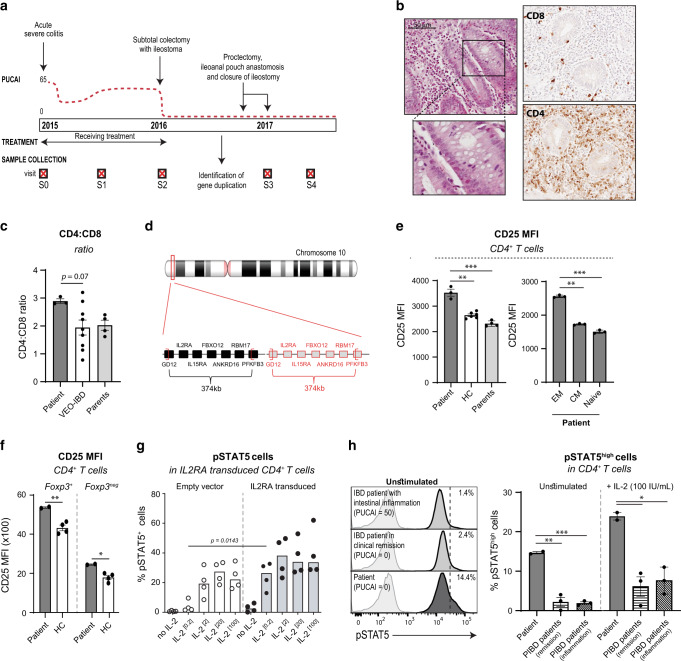
A 374 kb duplication on 10p15.1 including the *IL2RA* locus leads to intrinsically increased CD25 and enhanced IL-2 signaling in CD4^+^ T cells. **a** Timeline depicting the patient’s clinical course and time points of sample collection (denoted as visits S0-S4). PUCAI, pediatric ulcerative colitis activity index. **b** H&E staining on paraffin-embedded colonic tissue at the time of diagnosis (left, time point S0) and immunohistochemical detection of CD4 and CD8 in paraffin-embedded resected colonic tissue (right, time point S2). **c** Flow cytometric analysis of CD4:CD8 ratio in peripheral blood of the patient, her parents (time points S3 and S4), and VEO-IBD patients (*n* = 9). Median (5th to 95th percentiles) for the 2–5 year age category is 1.6 (0.9–2.9).^23^ **d** Localization of the patient’s duplication on chromosome 10. **e**–**g** Flow cytometric analysis of CD3, CD4, CD38, CD62L, CD45RA, CCR7, CD25, and/or Foxp3 expression was performed on peripheral blood from the patient, her parents (time points S3 and S4) and healthy adult controls (HC, *n* = 4–6). **e** CD25 expression (MFI) on total CD4^+^ T cells (left) and on effector memory (CD45RA^neg^CCR7^neg^), central memory (CD45RA^neg^CCR7^+^) and naive (CD45RA^+^CCR7^+^) CD4^+^ T cells in the patient (right). **f** CD25 expression on regulatory Foxp3^+^CD4^+^ T cells and Foxp3^neg^CD4^+^ T cells. **g** Frequency of pSTAT5-positive cells among control primary T cells transduced with retrovirus particles carrying full-length *IL2RA* or an empty vector upon stimulation with increasing concentrations of IL-2 (0.2 to 100IU/mL) (*n* = 4, *p* = 0.0143 at 0.2 IU/mL was calculated using Wilcoxon-Mann Whitney test). **h** PBMCs of the patient, PIBD patients with active intestinal inflammation (*n* = 3), and PIBD patients in remission (*n* = 3) were stimulated with IL-2 (100 IU/mL) for 15 min followed by quantification of STAT5 phosphorylation (pY694) in CD4^+^ cells by flow cytometry (visit S3). n.s., not significant, *p < 0.05, **p < 0.01, ***p < 0.001 using one-way ANOVA followed by the Bonferroni’s Multiple Comparison Test.

The patient is a girl who developed acute severe colitis at 2 years of age. She is the first child of non-consanguineous Caucasian parents and the only significant family history was left-sided ulcerative colitis (UC) of benign course diagnosed in her mother at 32 years (Fig. 1a). The child had no history of recurrent or opportunistic infections and diagnostic tests for cow’s milk protein allergy and celiac disease were negative. Endoscopic examination showed severe pancolitis with edematous and fragile mucosa and multiple erosions and pangastritis of mild intensity. In contrast, esophagus, duodenum, jejunum, and terminal ileum were macroscopically normal and there were no peri-anal or extra-intestinal manifestations. Histology showed chronic active colitis with crypt architectural distortion, destructive cryptitis, and crypt abscesses (Fig. 1b, left panel) and immunohistochemistry revealed an important lymphocytic infiltrate containing CD4^+^ but almost no CD8^+^ cells (Fig. 1b, right panel). Ileal biopsies at diagnosis showed a mixed inflammatory infiltrate and cryptitis that, in contrast to the colonic inflammation, resolved during immunosuppressive treatment. At diagnosis and before the start of immunosuppression, leukocyte count and absolute number of T, NK, and B lymphocytes in peripheral blood were within normal range.^23^ Serum concentrations of immunoglobulins were also normal. As the disease was refractory to standard immunosuppressive-, immunomodulatory-, and biological therapy, subtotal colectomy with temporary ileostomy was performed one year after initial diagnosis, followed 12 months later by proctectomy and ileoanal pouch anastomosis. Subtotal colectomy resulted in drastic clinical improvement without further need for immunosuppressive therapy (Fig. 1a). After surgery and without immunosuppression, despite normal absolute number of lymphocytes, the patient displayed a high CD4:CD8 ratio in peripheral blood when compared to age-matched VEO-IBD patients (Fig. 1c), a published age-matched reference population,^23^ adult and infant healthy controls, and adolescent PIBD with active disease (inflammation) or disease in remission (Fig. S1A). The patient’s clinical course and time points at sample collection are depicted in Fig. 1a and Table 1.

**Table 1:** Time points of sample collection.

Visit		Sample collection (peripheral blood and intestinal tissue)	Treatment	CRP (mg/L)	Calprotectin (μg/g)	PUCAI
February 2015	S0	Presentation with severe acute colitis. Diagnostic endoscopy with biopsies in small intestine and colon.	No treatment	19	2861 μg/g	55
August 2015	S1	Persistent disease activity using infliximab 10 mg/kg every 4 weeks and azathioprine 25 mg/day. Received blood sample.	Infliximab 5 mg/kg/day per 4 weeks, Azathioprine 1dd30 mg, Allopurinol 1dd50 mg, Cholecalciferol 1dd800IE, Omeprazol 1dd10 mg, Cotrimoxazol 1dd30mg	<3	ND	45
December 2016	S2	Subtotal colectomy with ileostoma. Received blood sample and intestinal tissue of patient.	Infliximab 5 mg/kg/day per 4 weeks (last infusion on 21^st^ of December 2015), Azathioprine 1dd30 mg, Allopurinol 1dd50 mg, Cholecalciferol 1dd800IE, Omeprazol 1dd10 mg, Vancomycin 2dd250 mg, Gentamicin 2dd50 mg	209	ND	55
January 2017	S3	Closure of double-barreled ileostomy, one month protectomy and ileoanal pouch anastomosis. No symptoms of intestinal disease. Received blood samples of patient and parents.	No immunosuppressive treatment. No recent antibiotic use.	ND	ND	0
August 2017	S4	Regular visit at outpatient clinic. No symptoms of intestinal disease. Received blood samples of patient and parents.	No immunosuppressive treatment. No recent antibiotic use.	ND	ND	0

*CRP* C-reactive protein, *PUCAI* pediatric ulcerative colitis activity index, *dd* daily dosis, *ND* not determined.

To identify a possible underlying genetic defect, the patient was screened for known mutations associated with VEO-IBD using targeted next-generation sequencing (TNGS) as described.^24^ DNA from the patient and both parents were next analyzed by whole-exome sequencing (WES, Fig. S1B). TNGS and WES failed to reveal any single gene mutation. Yet, increased DNA copy number was suggested by an excess in the number of reads derived from *IL2RA* encoding exons in the patient compared to other individuals simultaneously tested by TNGS or compared to the parents in WES analysis. Further analyses using array comparative genome hybridization (CGH, Fig. S1C), revealed a 374 kb duplication of the 10p15.1 chromosomal region, including the *IL2RA* and *IL15RA* genes (Fig. 1d, Table 2). CGH analyses were normal in both parents, confirming the de novo origin of the duplication.

**Table 2:** Genes involved in duplication on the 10p15.1 chromosomal region.

Gene symbol	Gene name	Function
*IL2RA*	IL-2 receptor alpha subunit	The IL-2 receptor alpha (IL2RA) and beta (IL2RB) chains, together with the common gamma chain (γ), constitute the high-affinity IL-2 receptor.
*IL15RA*	IL-15 receptor alpha subunit	The IL-15 receptor alpha subunit specifically binds interleukin 15 (IL-15) with high affinity. The receptors of IL15 and IL2 share two subunits, IL2R beta, and the common γ chain.
*FBXO18*	F-box protein, helicase 18	Constitutes one of the four subunits of an ubiquitin-protein ligase complex called SCFs (SKP1-cullin-F-box), which functions in phosphorylation-dependent ubiquitination.
*ANKRD16*	Ankyrin repeat domain-16	An ANK repeat is a protein containing at least one ANK repeat, a conserved domain of approximately 33 amino acids, that was originally identified in ankyrin. Associated with protein-protein interactions.
*RBM17*	RNA binding motif protein 17	Part of spliceosome complex and functions in the second catalytic step of mRNA splicing.

To define whether increased *IL2RA* and/or *IL15RA* expression may play a role in the patient’s intestinal disease, mRNA expression was compared in colonic tissue resected from the patient and from treatment-resistant PIBD patients. Strikingly, *IL2RA* (Fig. S1D) but not *IL15RA* (Fig. S1E) mRNA expression was elevated in the patient’s colonic tissue compared to PIBD controls. We therefore focused our analyses on the possible role of IL-2R in modulating immune function and predisposing to colonic inflammation.

### Duplication of the *IL2RA* locus is associated with increased CD25 expression and activation of the IL-2 pathway in peripheral CD4^+^ T cells

As IL-2 has important roles in T-cell survival and proliferation,^25–27^ we hypothesized that the observed duplication may potentiate T-cell activation. Flow cytometric analysis of peripheral blood cells was first performed one year after colectomy, when the child was in clinical remission and without medication (Fig. 1a, visits S3–S4). While there was no change in the frequency of CD25^+^ T cells (Fig. S2A), the mean fluorescence intensity of CD25 was significantly increased in CD4^+^ and CD8^+^ T cells from the patient compared to T cells from her parents and healthy adult individuals (Fig. 1e, left panel, and S2B). In particular, CD25 expression was increased on circulating effector memory CD4^+^ T cells compared to central memory and naïve CD4^+^ T cells from the patient (Fig. 1e, right panel). In contrast, CD25 expression did not differ between naive, effector memory, and central memory CD4^+^ T cells from the patient’s parents and adult healthy individuals (data not shown). In the patient, CD25 expression was also increased on circulating regulatory CD4^+^Foxp3^+^ T cells and non-regulatory CD4^+^Foxp3^neg^ T cells compared to controls (Fig. 1f), but the frequency of regulatory CD4^+^Foxp3^+^ T cells remained unchanged (Fig. S2C). Altogether, these data suggested that the patient T cells may overexpress CD25 after antigen stimulation. In line with these results, high concentrations of soluble CD25 were detected in the patient’s plasma at multiple visits, including after colectomy when the patient was free of clinical symptoms and without medication (Fig. S2D, visits S1–S3).

One major signaling cascade downstream IL-2 involves the activation of Janus kinase 3 (JAK3) and the subsequent phosphorylation of signal transducer and activator of transcription 5 (STAT5).^11^ To assess the impact of CD25 overexpression on STAT5 phosphorylation, human primary T cells derived from a healthy control were transduced with retrovirus particles carrying full-length CD25 or an empty vector. Positively transduced CD4^+^ T cells were selected based on LNGFR expression (Fig. S3A, B), and stimulated with increasing concentrations of IL-2. Strikingly, STAT5 activation was induced by very low IL-2 concentration (0.2 U/mL) in LNGFR^+^ CD4^+^ CD25^+^ CD4^+^ T cells carrying the full length CD25 but not in LNGFR^+^ CD4^+^ CD25^+^ CD4^+^ T cells transduced with the empty vector (Figs. 1g, S3C) indicating that overexpression of CD25 increased responsiveness to IL-2 in CD4^+^ T cells. STAT5 phosphorylation was next compared in peripheral blood CD4^+^ T cells from the patient (visit S3) and from pediatric IBD patients with active intestinal inflammation (“PIBD inflammation”) or in clinical remission (“PIBD remission”). A fraction of circulating CD4^+^ T cells from the patient was activated as evidenced by their increased STAT5 phosphorylation compared to circulating CD4^+^ T cells from pediatric IBD patients at baseline (Fig. 1h). Moreover, STAT5 phosphorylation after a 15-minute stimulation with exogenous IL-2 was significantly increased in the patient’s CD4^+^ T cells compared to T cells from PIBD controls (Fig. 1h, Fig. S3D).

Overall, these data led us to conclude that de novo duplication of *IL2RA* gene promoted CD25 expression on circulating effector memory CD4^+^ T cells and enhanced their responsiveness to IL-2, as recapitulated in vitro.

### CD4^+^ T cells of the patient with duplication of *IL2RA* display increased responsiveness to T-cell receptor (TCR) stimulation

**Fig. 2:**
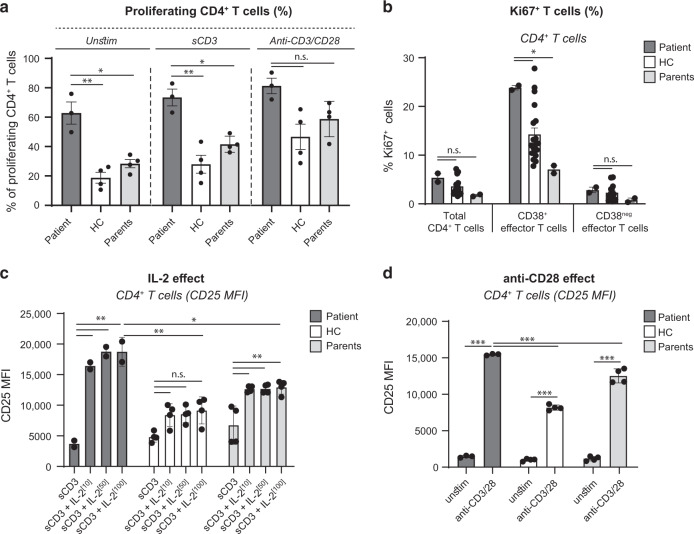
CD25 overexpression is associated with increased response to IL-2. **a** Patient PBMCs (visits S3 and S4) and healthy adult control (*n* = 4) were stimulated with anti-CD3 (500 ng/ml) or anti-CD3/CD28 beads (bead-to-cell ratio 1:2) for 48 h. The percentage of proliferating CD4^+^ T cells was analyzed by CellTrace Violet dilution. **b** Frequencies of Ki67^+^ cells gated on CD4^+^ T cells, CD62L^neg^CD38^+^CD4^+^ T cells and CD62L^neg^CD38^neg^CD4^+^ T cells. Data are mean ± SEM (healthy adult controls, *n* = 19). **c** PBMCs were stimulated with anti-CD3 (500 ng/ml) in the absence or presence of IL-2 (10, 50, or 100 IU/ml). After 48 h, CD3, CD4, and CD25 expression were determined by flow cytometry. **d** PBMCs were stimulated with anti-CD3/CD28 beads (bead-to-cell ratio 1:2). CD25 expression on CD4^+^ T cells was analyzed at 48 h. Data are mean ± SEM (*n* = 4). Representative of two independent experiments (time points S3 and S4); n.s., not significant, *p < 0.05, **p < 0.01, ***p < 0.001 using one-way ANOVA followed by the Bonferroni’s Multiple Comparison Test.

As indicated above, cellular infiltration in the inflamed colon of the patient consisted predominantly of CD4^+^ T cells (Fig. 1b), suggesting that enhanced IL-2 responsiveness may preferentially promote the expansion of CD4^+^ T cells of mucosal origin. Analyses of CFSE dilution demonstrated that patient CD4^+^ T cells contained a significantly higher frequency of spontaneously dividing cells than CD4^+^ T cells from controls (Fig. 2a). Moreover, the patient’s CD4^+^ T-cell proliferation remained enhanced upon anti-CD3 and anti-CD3/CD28 activation (Fig. 2a). To investigate whether the increased CD4:CD8 ratio observed in peripheral blood may indeed reflect the expansion of mucosal CD4^+^ T cells, we analyzed the expression of the proliferation marker Ki67 in CD62L^neg^CD38^+^CD4^+^ T cells which are enriched in gut-homing T cells specific for mucosal antigens.^28,29^ In keeping with our hypothesis, circulating CD62L^neg^CD38^+^CD4^+^ T cells, but neither total CD4^+^ T cells nor CD62L^neg^CD38^neg^CD4^+^ T cells, contained higher frequencies of proliferating Ki67^+^ cells in the patient than in controls (Fig. 2b). We therefore concluded that the increased Ki67 expression likely reflected enhanced in vivo proliferative rate of CD4^+^ T cells of mucosal origin.

We next examined whether the increased proliferative rate of circulating CD4^+^ T cells in the patient with *ILR2A* duplication may be explained by their enhanced responsiveness to IL-2. In the absence of TCR ligation, the addition of exogenous IL-2 induced a comparable increase in CD25 expression in CD4^+^ and CD8^+^ T cells from the patient and from controls (data not shown). However, following ligation of CD3, IL-2 potentiated significantly more CD25 upregulation in CD4^+^ T cells from the patient than from controls (Fig. 2c). Moreover, a low concentration of exogenous IL-2 (10 IU/ml) was sufficient to cause a strong increase in CD25 expression in CD3-stimulated CD4^+^ T cells from the patient (Fig. 2c). Ligation of CD3 together with CD28 also caused a more pronounced up-regulation of CD25 in CD4^+^ T cells from the patient than from controls (Fig. 2d). Of note, percentages of live cells were similar between patient and control cell cultures (data not shown). Comparable results were observed in CD8^+^ T cells but the differences between patient and control cells were less prominent (Fig. S4), a result agreeing with the higher absolute numbers of CD4^+^ T cells in all culture conditions (data not shown) as well as in vivo in peripheral blood and colonic tissue of the patient.

Taken together, these data show how increased T-cell responsiveness to IL-2 in the patient with *IL2RA* duplication can amplify the proliferative response of CD4^+^ T cells to antigenic stimulation and license the expansion of peripheral T cells initially activated in the antigen-rich intestinal environment.

### Colonic CD4^+^ IEL and LPL of the patient with IL2RA duplication show increased proliferation and activation compared to T cells of treatment-resistant pediatric-onset IBD patients

To investigate whether and how increased T-cell responsiveness to IL-2 may predispose to colonic inflammation, T-cell infiltration and activation were compared in colonic tissue resected from the patient with the *IL2RA* duplication and from eight pediatric-onset treatment-resistant IBD patients (four UC patients, four Crohn’s disease (CD) patients, denoted as “PIBD resection controls”). In the patient, colonic inflamed biopsies were also compared to non-inflamed duodenal tissue obtained during the initial assessment at diagnosis.

**Fig. 3:**
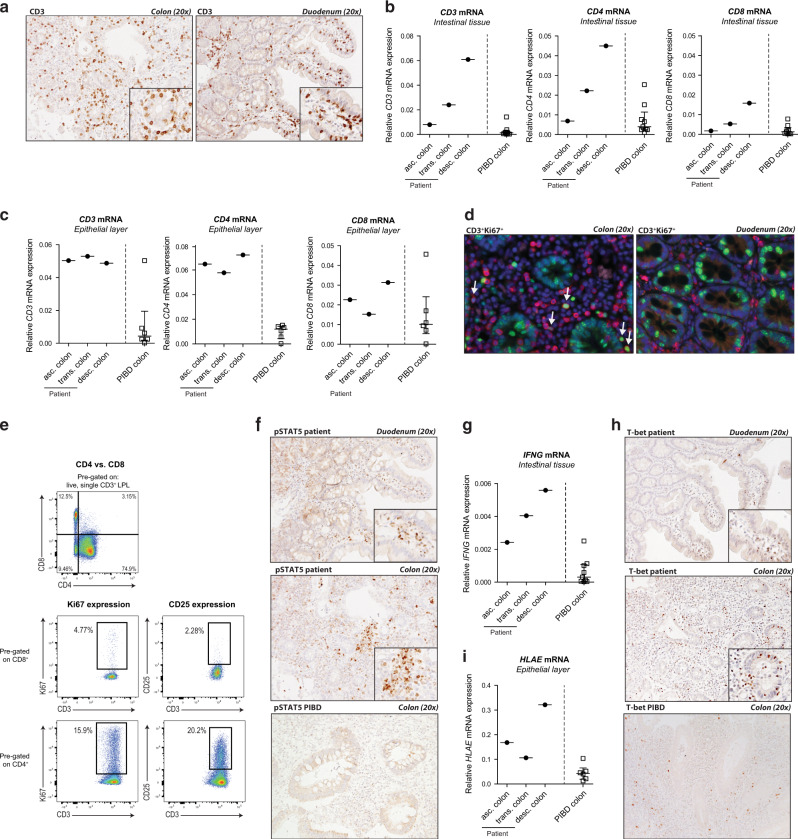
Increased proliferation and activation of colonic CD4^+^ IEL and LPL in the patient with IL2RA duplication compared to treatment-resistant pediatric-onset IBD. **a**, **f**, **h** Representative immunohistochemical staining for CD3, pSTAT5, and Tbet in paraffin-embedded resected inflamed colonic tissue (visit S2) and paraffin-embedded tissue of the unaffected duodenum from the patient at time of diagnosis (visit S0) and PIBD control. **b** *CD3*, *CD4,* and *CD8* mRNA and **g** *IFNG* mRNA expression in total resected colonic tissue of the patient and PIBD controls. **c** *CD3*, *CD4,* and *CD8* mRNA and (**i**) *HLAE* mRNA expression in the epithelial layer isolated from resected colonic tissue of the patient and PIBD controls. **d** Representative immunofluorescent double staining of paraffin-embedded resected colonic tissue of the patient. Green = Ki67, red = CD3, blue = 4′,6-diamidino-2-phenylindole (DAPI) nuclear staining. **e** LPL were isolated from the inflamed colonic tissue of the patient. Frequencies of CD4^+^ and CD8^+^ cells in live CD3^+^ LPL, and Ki67 and CD25 expression by CD4^+^ and CD8^+^ LPL were analyzed by flow cytometry.

As already indicated and as depicted in Fig. 1b, the patient’s colon was strongly infiltrated by CD3^+^ T cells in both *lamina propria* and epithelium (Fig. 3a). In order to precisely compare the density and nature of the colonic T-cell infiltrate in the patient and in the inflamed controls, mRNA encoding *CD3*, *CD4*, and *CD8* were quantified by RT-PCR. Expression of *CD3* and *CD4* but not *CD8* mRNA was strikingly higher in whole colonic tissue derived from the patient than from PIBD resection controls (Fig. 3b). Comparable results were obtained when mRNA was extracted from the epithelial layer (Fig. 3c), which normally contains predominantly CD8^+^ T cells,^30^ sustaining evidence that CD4^+^ T cells were more particularly prone to expand in the patient. Accordingly, immunohistochemistry showed a marked increase in the number of proliferating Ki67^+^CD3^+^ T cells in the *lamina propria* and epithelium of the patient’s colon compared to inflamed colon resected from PIBD controls (Figs. 3d, S5A–C). Flow cytometry further indicated that most Ki67^+^ lamina propria lymphocytes (LPL) isolated from the patient’s colon were CD4^+^ (Fig. 3e). Consistent with the hypothesis that proliferation of colonic T cells resulted from their increased responsiveness to IL-2, many CD4^+^ but no CD8^+^ colonic LPL from the patient expressed CD25 (Fig. 3e). Moreover, multiple clusters of cells displaying nuclear pSTAT5 were observed in the patient’s colon while they were rare in colonic tissues resected from PIBD controls (Figs. 3f, S5B, C). Of note, the numbers of proliferating CD3^+^Ki67^+^ cells and pSTAT5^+^ cells were very low in the histologically normal duodenum of the patient (Figs. 3d, S5D), suggesting that expansion of colonic T cells may be driven by the microbiota that is considerably more abundant in the distal intestine than in duodenum.

In order to define how the CD4^+^ T cells, which proliferate in the patient’s colon, may contribute to tissue damage, we next examined mRNA expression of effector molecules and inflammatory cytokines. Confirming the lack of colonic CD8^+^ T-cell activation, there was no change in the amounts of mRNA encoding Natural Killer Group 2D *(NKG2D)*, a NK receptor expressed by CD8^+^ IEL, nor in mRNA encoding granzyme B *(GZMB1)* and perforin *(PRF1)*, two effector molecules mediating the cytolytic capacity of IEL, in the patient when compared to PIBD controls (data not shown). Also, very few IL-17^+^ cells were detected in the patient’s affected colonic tissue (data not shown). In contrast, *IFNG* and *IL21* mRNA were increased in the inflamed colonic tissue of the patient compared to inflamed PIBD resection controls (Figs. 3g, S6A, b) and numerous T-bet^+^ and IL-21^+^ cells were detected by immunohistochemistry in the lamina propria and epithelial layer of the colon (but not of duodenum; Figs. 3h, S6C). In agreement, IFNγ^+^- and IL-21^+^ were detectable by flow cytometry in lamina propria CD4^+^ T cells isolated from the patient’s colon (Fig. S6D). Finally, mRNA encoding the non-classical MHC-I molecule *HLAE* (Fig. 3i), was higher in the patient’s colonic epithelial layer compared to pediatric IBD controls, likely reflecting the increased local production of IFNγ.

Altogether, these data indicated that increased T-cell responsiveness to IL-2 in the patient with *IL2RA* duplication licensed expansion and activation of colonic CD4^+^ T cells expressing T-bet and secreting large amounts of IFNγ and IL-21.

### Increased IL-2 signaling enhances TCR-driven IFNγ production in CD4^+^ T cells of the patient and this effect is reversed by JAK1/3 inhibition

**Fig. 4:**
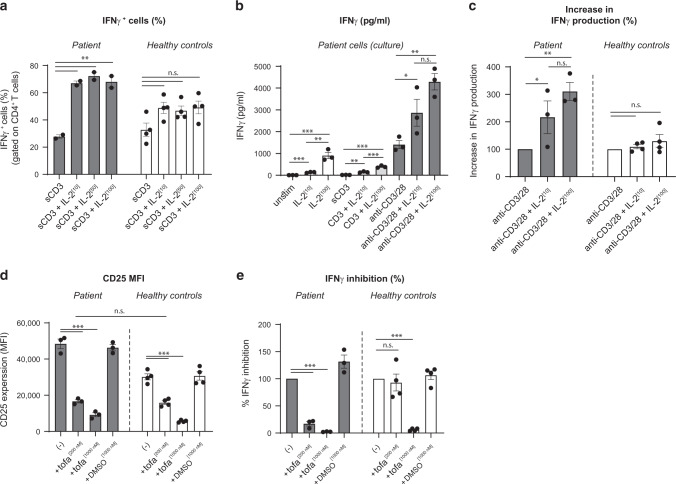
Increased IL-2 signaling enhances IFNγ production and is reversed by JAK1/3 inhibition. **a**–**c** PBMCs of the patient and healthy adult controls were stimulated with anti-CD3 (500 ng/ml) or anti-CD3/anti-CD28 beads (bead-to-cell ratio 1:2) in the absence or presence of IL-2 (1, 50, or 100 IU/ml) for 48 h. **a** Percentage of IFNγ-expressing CD4^+^ T cells were analyzed by flow cytometry. **b** IFNγ secretion by patient cells was analyzed using ELISA. **c** IFNγ responses of patient and healthy adult donors are shown. The relative increase in IFNγ secretion between anti-CD3/CD28 and cultures with IL-2 is shown (considering the percentage of cytokine secretion upon anti-CD3/CD28 stimulation as 100%). **d**, **e** PBMCs of the patient and healthy adult controls were stimulated with anti-CD3/anti-CD28 beads (bead-to-cell ratio 1:2) in the absence or presence of tofacitinib (200 or 1000 nM) for 48 h. **d** CD25 expression on CD4^+^ T cells was analyzed by flow cytometry. **e** Supernatants were assayed for IFNγ using an ELISA. The relative difference in IFNγ secretion between anti-CD3/CD28 and cultures with tofacitinib is shown (considering the percentage of cytokine secretion upon anti-CD3/CD28 stimulation as 100%). Data are mean ± SEM (adult healthy controls, *n* = 4); n.s., not significant, *p < 0.05, **p < 0.01, ***p < 0.001 using one-way ANOVA followed by the Bonferroni’s Multiple Comparison Test.

To assess if this high IFNγ response was related to the increased CD25 expression and IL-2 signaling observed in the patient’s T cells, we next compared the effect of IL-2 on IFNγ production in PBMC from the patient and from controls. A significant enhancing effect of IL-2 on the frequency of IFNγ-producing CD4^+^ T cells was observed in the patient’s PBMC stimulated with anti-CD3, but not in the control PBMC (Fig. 4a). Along the same line, exogenous IL-2 potentiated the secretion of IFNγ induced by CD3 or anti-CD3/CD28 stimulator beads in PBMC of the patient but not of controls (Fig. 4b, c). To further demonstrate the role of IL-2 signaling in potentiating IFNγ secretion by patient’s T cells, we used the JAK1/3 inhibitor tofacitinib to inhibit IL-2 signaling. As shown in Fig. 4d, tofacitinib restored normal CD25 expression (Fig. 4d) and significantly reduced IFNγ secretion by patient CD4^+^ T cells (Fig. 4e). This result was achieved at a suboptimal concentration of tofacitinib (200 nM), which had no effect on IFNγ secretion by control PBMC (Fig. 4e). A higher concentration of tofacitinib (1000 nM) equally reduced IFNγ secretion in PBMC from healthy controls and from the patient (Fig. 4e). These data support the view that the enhanced IFNγ response observed in the patient’s colon resulted from increased CD25 expression and subsequent enhanced IL-2 signaling.^31,32^

### Low incidence of *IL2RA* locus microduplications

Despite the young age and very severe IBD, a subtotal colectomy elicited a drastic clinical improvement without further need of immunosuppressive treatment suggesting that *IL2RA* duplication may predispose to IBD but that additional factors are required to develop disease. To formally establish a causal relation between the *IL2RA* locus duplication and intestinal inflammation a search for similar 10p15.1-duplications in public control and patient databases was performed (Table 3). There were no reports for similar 10p15.1-duplications in the control population. In the patient databases Decipher and ClinVar 19 and 13 cases were reported respectively. Most of these duplications were very large in comparison to the 374 kb microduplication in our study. No “inflammation of the large intestine” or “Crohn’s disease” was reported in any of these cases. Via a personal communication we found 1 patient with a smaller 10p15.1-duplication containing the *IL2RA* locus only. No IBD phenotype was reported for this patient. Together, these data suggest a relatively low incidence of the *IL2RA* locus microduplication which hampers investigations to assess causality of the genetic aberration in predisposing to VEO-IBD.

**Table 3:** Search for similar 374 kb 10p15.1-duplications including *IL2RA* in public databases (excluded small duplications (1 and 5 bp), duplications extending into 10q-arm, and size uncertain.

	Database	Gain including *IL2RA*	Total number of entrees in database	Size duplication median (range)	Reported phenotype
Control population	WTCCC_3000^a^	0	2931	n.a.	n.a.
Patient database	Database of Genomic Variants (DGV)^b^	0	>7,000,000	n.a.	n.a.
	Decipher^c^ (no comparable duplications, selected hits limited to 10p-arm)	*n* = 19	>27,000	12.9 Mb (2.9–32.5)	*n* = 8 no phenotype entered *n* = 11 no inflammation of the large intestine OR Crohn’s disease
	ClinVar^d^	*n* = 13	>493,240	12.7 Mb (0.6–26.4)	*n* = 2 no phenotype entered *n* = 11 no inflammation of the large intestine OR Crohn’s disease
	Dutch consortium CNV database^e^	*n* = 1		174 Kb (including IL2RA, excluding IL15RA)	*n* = 1 no IBD reported

^a^Wellcome Trust Case Control Consortium (https://www.wtccc.org.uk/).

^b^Database of Genomic Variants (http://dgv.tcag.ca/dgv/app/home).

^c^Decipher (https://decipher.sanger.ac.uk/).

^d^NCBI ClinVar (https://www.ncbi.nlm.nih.gov/clinvar/).

^e^Personal communication.

## Discussion

To our knowledge, this is the first description of a patient with an *IL2RA* locus duplication presenting with treatment-resistant colitis at 2 years of age. The patient’s colon, but not the unaffected small intestine was infiltrated with proliferating CD3^+^, predominantly CD4^+^ T cells, and contained numerous T-bet^+^ cells expressing high levels of *IFNG* mRNA. After subtotal colectomy and during complete clinical disease remission, CD25 expression was increased in circulating effector memory CD4^+^ T cells and on-going activation of peripheral CD4^+^ T cells was evidenced by increased STAT5 phosphorylation and proliferation. In keeping with their enhanced expression of CD25, peripheral CD4^+^ T from the patient showed increased responsiveness to IL-2, a functionality that was recapitulated in vitro by overexpression of *IL2RA* in healthy control CD4^+^ T cells. Increased IL-2 responsiveness potentiated their production of IFNγ after TCR stimulation. Conversely, inhibiting IL-2 signaling with the JAK1/3 inhibitor tofacitinib restored normal CD25 expression and ablated TCR-induced IFNγ secretion in the patient’s T cells. Altogether, these results indicate that duplication of the *IL2RA* gene causes increased upregulation of CD25 in activated CD4^+^ T cells, which, as a consequence, undergo excessive stimulation in the antigen-rich environment of the colon and induce inflammation.

Our results showing that duplication of the *IL2RA* locus potentiates CD25 expression and IL-2 signaling are reminiscent of previous reports showing that CD25 surface expression on memory T cells is variable and can be correlated with haplotypes of the *IL2RA* region, conferring susceptibility to type I diabetes and multiple sclerosis.^33^ Along the same line, susceptibility to UC and CD associates with single nucleotide polymorphisms (SNPs) in the *IL2RA* locus^3^ although quantitation of CD25 surface expression on CD4^+^ T cells of individuals bearing this IBD-associated SNP has not yet been reported. Understanding the functional consequences of genetic variation in the *IL2RA* locus in IBD and determining associating disease patterns and therapy responsiveness may therefore have relevance for IBD.

As subtotal colectomy has now effectively induced clinical remission in the patient for more than 27 months without further need of medication but did not correct the increased levels of CD25 on T cells, we conclude that the *IL2RA* locus duplication alone is not sufficient to drive disease. As development of the microbial-host mutualism is a key immunological process in the intestine at a young age, we hypothesize that microbial stimulation and subsequent antigenic triggering of T cells may have initiated colonic disease. Accordingly, the diseased colon contained many proliferating CD3^+^Ki67^+^ cells with increased STAT5 phosphorylation, which were absent in the duodenum where the concentration in bacteria is considerably less. Despite elevated CD25 expression on both CD4^+^ and CD8^+^ T cells, infiltration of the colonic mucosa was dominated by CD4^+^ T cells, further suggesting that microbiota-derived antigen may have triggered the disease. Supporting this hypothesis, anti-microbial antibodies with a wide range of specificity were detectable in the patient’s plasma (data not shown). It is unclear why the therapy-resistant disease mostly persisted in colon. At the time of diagnosis the patient had ileal disease with a mixed inflammatory infiltrate and cryptitis which, in contrast to the colonic disease, had strongly diminished after intensive treatment with prednisolone, azathioprine, and anti-TNF. Interestingly, the cellular infiltrates in the affected colonic tissue comprised many T-bet^+^, IFNγ^+^, and IL-21^+^ cells, but only very few IL-17^+^ cells. These observations are in line with previous studies demonstrating that IL-2 signaling supports Th1 responses, induces *Tbx21* and IFNγ^31,34^ but may inhibit Th17 responses.^35,36^

The patient’s duplication consisted of a 374 kb genomic segment including the *IL2RA* gene, the *IL15RA* gene, and three additional genes (see Table 2 for all genes and their functions contained in the duplication). To date, none of the additional three genes (*FBXO18*, *ANKRD16,* and *RBM17*) have been reported to be involved in immune responses. Moreover, although the genes *GDI2* and *PFKFB3* contain breakpoints framing the duplication allowing for a loss of function, further analyses of this probability suggested that loss of function on one allele should be tolerated. In contrast, it cannot be formally excluded that, besides functional changes in the CD25 pathway, increased IL-15 signaling may have participated in the patient’s disease. However, no enhanced *IL15RA* mRNA could be detected in the inflamed colon compared to PIBD controls and cell numbers of leukocyte populations preferentially maintained by IL-15, such as NK cells and CD8^+^ memory T cells,^37,38^ were increased neither in the patient’s peripheral blood nor in colonic tissue. Moreover, in vitro stimulation assays with IL-15 did not reveal increased IL-15 receptor function. Of note, IL-15 signaling in T cells mainly depends on a signaling module consisting of the IL-2Rβ chain and of the common γ chain that is shared with IL-2. Yet, and in contrast with IL-2, IL-15 binds the βγ_c_ module with high avidity and does not require IL-15RA for downstream activation of the JAK/STAT pathway and IL-15RA seems mainly involved in vivo in the transpresentation of IL-15/IL-15Rα complexes to lymphocytes.^39^

Establishing direct causality between the genetic aberration and VEO-IBD was proven difficult as very few similar microduplications of the *IL2RA* locus have been found in genetic control and patient databases. For patients with much larger duplications including the *IL2RA* locus it is difficult to assess whether IBD would have been reported relative to their other severe complaints and, if so, whether genetic changes in the large numbers of other duplicated genes would compensate for functional changes in the *IL2RA* locus.

Although steroids and immunomodulators are still widely used in the treatment of IBD, small molecule inhibitors such as tofacitinib that specifically inhibit the JAK1 and JAK3 pathways are now becoming available. Recently, tofacitinib has been approved by the U.S. Food and Drug Administration and European Medicines Agency for the treatment of patients with moderate to severe UC. In keeping with our previous report that the JAK1/2 inhibitor ruxolitinib effectively inhibited enterocolitis in a patient with STAT3 gain of function mutation,^40^ our findings strengthen the argument that a subgroup of IBD patients may benefit from a tight control of the JAK/STAT pathway to inhibit intestinal inflammation. Hence, it is crucial to develop a robust set of immunological assays to predict and monitor therapeutic success at an individual level. Although, the here discovered pattern of hyper-CD25 associated inflammation was not dominant in the eight control pediatric-onset IBD patients used in this study, future studies are aimed to identify IBD patients with a similar pattern of hyper-IEL, IFNγ, and pSTAT5 positive lesions. This may uncover a specific subgroup of patients with predominant CD4^+^ T-cell driven disease, who might benefit from JAK/STAT signaling targeting therapies.

Taken together, our data show how increased CD25 expression and enhanced IL-2 signaling can amplify the proliferative and inflammatory response of CD4^+^ T cells to antigenic stimulation, licensing expansion of CD4^+^ T cells activated in the antigen-rich intestinal environment. Such a response can associate with colonic inflammation and contrasts with the severe autoimmune enteropathy that can develop in patients lacking CD25,^8^ highlighting the importance of tightly controlled of IL-2 signaling to preserve intestinal homeostasis.

## Methods

### Patients

Peripheral blood was obtained from the VEO-IBD patient described below, her parents, a cohort of VEO-IBD patients (*n* = 9), pediatric-onset IBD (PIBD) patients in clinical remission (*n* = 4), pediatric IBD patients with active intestinal inflammation (*n* = 4) and adult healthy controls (*n* = 15). Small intestinal and colonic biopsies were obtained from the VEO-IBD patient at the time of diagnosis. Specimens of resected colonic tissue were obtained from the VEO-IBD patient and PIBD patients (*n* = 8) refractory to conventional and biological immunosuppressive therapy. The Medical Ethical Committee of the Erasmus University Medical Center-Sophia Children’s Hospital Rotterdam approved this study (METC 2007-335). Written informed consent was obtained from every patient and parents before study inclusion.

### Genetic analysis

TNGS and whole-exome sequencing (WES) were performed as described previously.^24,41^ Array-comparative genome hybridization (CGH) of DNA extracted from peripheral blood cells was performed on an Agilent 60 K oligo- nucleotide microarray (Agilent Technologies, Santa Clara, California, USA).

### Cell isolation and cultures

Venous blood was collected in EDTA tubes and PBMCs were isolated using a Ficoll-Hypaque gradient according to standard protocol (Axis-Shield Diagnostics, Dundee, UK). PBMCs were labeled with CellTrace Violet (ThermoFisher Scientific, Bleiswijk, the Netherlands) and were stimulated with phytohemagglutinin (PHA, 5 µg/ml, ThermoFisher Scientific), ConA (10 µg/ml), anti-CD3 (0.5 µg/ml, Sanquin, Amsterdam, the Netherlands) or anti-CD3/CD28 stimulator beads (0.5 bead per PBMC) with or without recombinant human IL-2 (1, 10 or 100 IU/ml, R&D Systems, Minneapolis, MN, USA) or IL-15 (1, 10 or 100 µg/ml, R&D Systems) for the indicated time-points. In some experiments, tofacitinib (Pfizer, New York, NY, USA; 200 or 1000 µM) was added to the anti-CD3/CD28 stimulated conditions. Cells were cultured in Iscove’s modified Dulbecco’s medium (ThermoFisher Scientific) supplemented with heat-inactivated fetal calf serum, Glutamax (ThermoFisher Scientific), 2-mercaptoethanol, penicillin, and streptomycin. For retroviral viral transduction, PBMCs from a healthy donor were isolated and T cell blasts were generated by culturing PBMCs with 1 µg/ml PHA (Sigma-Aldrich, Zwijndrecht, The Netherlands) for 72 h in RPMI-1640 medium supplemented with 10% human serum (#H4522, Sigma-Aldrich) and expanded in culture with 100 IU/ml recombinant human IL-2 (R&D Systems).

### Plasmids, retrovirus production, transduction, and pSTAT5 activation

The IL2RA cDNA was subcloned from pEGFP-N1 (#86055, Addgene, Watertown, MA, USA) into a pLZRS-IRES-ΔNGFR (#72930, Addgene) via Dra1/EcoR1 sites and the tag EGFP was cut-out. The pLZRS-IRES-ΔNGFR (empty vector) and pLZRS-IRES-IL2RA-ΔNGFR retroviral vectors were produced as previously described.^42^ Phoenix-AMPHO packaging cells (ATCC® CRL-3213™) were transfected with 10 μg of pLZRS-IRES-ΔNGFR or pLZRS-IRES-IL2RA-ΔNGFR by lipofectamine 2000 (Life technologies, Carlsbad, CA, USA). ΔNGFR positive cells were selected by puromycin (ThermoFisher Scientific), at a concentration of 2 μg/ml. Phoenix-AMPHO cells expressing either pLZRS-IRES-ΔNGFR or pLZRS-IRES-IL2rA-ΔNGFR were cultured for 24 h, the supernatant was harvested and the retroviral particles were concentrated in a ratio 3:1 with Retro-X concentrator (Takara, Clontech, Saint-Germain-en-Laye, France). T cell blasts at 1 × 10^6^ cells /ml were transduced by centrifugation at 800 × *g* for 30 min in a retronectin-coated plate (Takara, Clontech). Seven days later, the yield of transduction was evaluated by assessing LNGFR positive cells by flow cytometry. At day 7 post transduction, cells were IL2-starved over 36 h, and stimulated with increasing dosages of IL-2 (0.2 IU/ml to 100 IU/ml) for 20 min. IL-2-induced STAT5 phosphorylation (pSTAT5) was then assessed by flow cytometry.

### Flow cytometry

PBMCs were stained for flow cytometry ex vivo or after in vitro culture using monoclonal antibodies against CD3 (UCHT1, BD Biosciences, Franklin Lakes, NJ, USA; HIT3a, Biolegend, San Diego, CA, USA), CD4 (SK3, BD), CD8 (SK1, BD), CD38 (HIT2, BD), CD62L (DREG-56, Biolegend), CD25 (2A3, BD), CD45RA (HI100, BD), CD45RO/RPE (UCHL1, Agilent, Santa Clara, CA, USA), TIGIT (MBSA43, eBioscience, Bleiswijk, the Netherlands) and CCR7 (15053, R&D Systems). Intracellular staining was performed with the Foxp3 fixation and permeabilization staining buffer kit, according to manufacturer’s protocol (eBioscience), followed by staining with anti-Foxp3 (236A/E7 or PCH101, eBioscience), anti-Helios (22F6, Biolegend), or anti-Ki67 (20Raj1, eBioscience) and the appropriate isotype controls (eBioscience).

For intracellular phosphorylated STAT5 staining, PBMCs were rested overnight at 37 °C and stained for CD4 (RPA-T4, BD; RPA-T4, Sony Biotechnology), CD8 (SK1 Sony Biotechnology), CD25 (M-A251, Sony Biotechnology), pSTAT5 (pY694, BD), and LNGFR (CD271) (REA648, Miltenyi Biotec, Bergisch Gladbach, Germany); Fixable Live/dead (ThermoFisher Scientific) or the appropriate isotype control according to the manufacturer’s protocol (Alternative protocol 1, Fix-stain-perm, BD Phosflow). In several conditions, cells were stimulated with IL-2 for 15–20 min prior to analysis. Cells were analyzed using the FACS Canto II and FlowJo software (BD).

### Cytokine and soluble CD25 analysis

Cytokine concentrations in cell supernatants or plasma were analyzed using an enzyme-linked immunosorbent assay set for IFNγ (eBioscience) according to the manufacturer’s instructions. Soluble CD25 levels in plasma were determined using the human CD25/IL-2R alpha Quantikine ELISA kit according to the manufacturer’s instructions (R&D Systems).

### Epithelial cell and LPL isolation

A specimen of total intestinal tissue was stored for immunohistochemistry (4% paraformaldehyde) and RNA isolation (RNA later, Sigma-Aldrich) prior to incubation. Next, total intestinal tissues were incubated in 0.15% dithiothreithol (DTT)/HBSS for 15 min and in 1 mM EDTA/HBSS for 30 min at 60 rpm and 37 °C. Epithelial layers were stored in RA1 buffer with DTT (1:100, Macherey-Nagel, Bethlehem, PA, USA) for RNA isolation. Intestinal specimens without epithelial fractions were incubated in a digestive solution consisting of 10% FCS, 25 mM HEPES, 100 U/ml penicillin streptomycin, 30 μg/ml gentamycin, 0.5 μg/ml fungizone, 0.1 mg/ml collagenase III, and 1 mg/ml DNase for 60 min at 37 °C. In several experiments, LPL were stimulated for 5 h with phorbol 12-myristate 13-acetate (PMA, 0.02 μg/ml, Sigma-Aldrich) and ionomycin (0.5 μg/ml, Sigma-Aldrich) in the presence of Brefeldin A (3 μg/ml eBioscience) for the last 2 h and subsequently analyzed for intracellular cytokine expression.

### RNA isolation and quantitative PCR

Total RNA was extracted from total intestinal tissues or epithelial layers using the Nucleospin RNA II or XS kit (Macherey-Nagel) and reverse transcribed into cDNA using the SensiFAST cDNA synthesis kit (Bioline, London, UK). Real-time quantitative PCR was performed using SYBR Green on a AbiPrismR 7900 Sequence Detection system (Applied Biosystems, Foster City, CA, USA). The relative expression to *GAPDH* for each gene was measured as 2^(−ΔCt)^. Primer sets used were: *GAPDH*, Fw: 5′-GTCGGAGTCAACGGATT-3′, Rv: 5′-AAGCTTCCCGTTCTCAG-3′; *CD3*ε, Fw: 5′-GGGCAAGAGTGTGTGAGA-3′, Rv: 5′-CGGGAGGCAGTGTTCT-3′; *CD4*, Fw: 5′-GGCATCTTCTTCTGTGTCA-3′, Rv: 5′-CCTCGTGCCTCAAATG-3′; *CD8α*, Fw: 5′-GAACCGAAGACGTGTTTG-3′, Rv: 5′-CGCCCCCACTAAAATAAT-3′-3′; *HLAE*, Fw: 5′-TCCGAGCAAAAGTCAAAT -3′, Rv: 5′- GCCAGGTCAGTGTGATCT-3′; *NKG2D*, Fw: 5′- AGCCAGGCTTCTTGTATGT-3′, Rv: 5′-TTCCTGGCTTTTATTGAGAT -3′; *MICA*, Fw: 5′-ATGGGAATGGAACCTACC-3’, Rv: 5′-TCTGCCAATGACTCTGAAG-3′; *GZMB*, Fw: 5′-TGGGGAAGCTCCATAAA -3′, Rv: 5′- GGGCCTTGTTGCTAGG-3′; *PRF1*, Fw: 5′-GAGCCTCGGTGAAGAGA-3′, Rv: 5′-GCGCTTGCACTCTGAG-3′; *IFNG*, Fw: 5′- CCAGGACCCATATGTAAAAG-3′, Rv: 5′-TGGCTCTGCATTATTTTTC-3′; *IL21*, Fw: 5′-AAGGCCCAACTAAAGTCAG-3′, Rv: 5′- AGGGCATGTTAGTCTGTGTT-3′; *CD25*, Fw: 5′-GCCGTCCTGAGAGTGAG-3′, Rv: 5′-TTCCCGGCTTCTTACC-3′; *IL15RA*, Fw: 5′-GCCGCCAGGTGTGTAT-3′, Rv: 5′-TGGTCCCCCAAGTCAC-3′.

### Immunohistochemistry

Paraffin-embedded biopsies were sectioned, deparaffinized, and endogenous peroxidase activity was quenched with 3% H_2_O_2_ in PBS for 20 min. Antigen retrieval was performed by microwave treatment in citrate buffer (10 mM, pH 6.0) or EDTA buffer (1 mM, pH 8.0). The sections were blocked for 1 h in 10% normal human serum plus 10% normal goat, rabbit or horse serum diluted in 10 mM Tris, 5 mM EDTA, 0.15 M NaCl, 0.25% gelatin, 0.05% Tween-20, pH 8. Antibody incubation was performed overnight at 4 °C using anti-Ki67 (monoclonal rabbit, D2H10, Cell Signaling Technology, Danvers, MA, USA), anti-CD3 (polyclonal rabbit, DakoCytomation), anti-pSTAT5 (Tyr694, C11C5, monoclonal rabbit, Cell Signaling Technology), anti-Tbet (4B10, monoclonal mouse, eBioscience), anti-IL-21 (polyclonal rabbit, Lifespan Biosciences, Seattle, WA) or matching isotype antibodies. Immunoreactive sites were detected with biotinylated secondary antibodies using the Vectastain ABC Elite Kit (Vector Laboratories, Burlingame, CA, USA) and 3,3′-diaminobenzidine tetrahydrochloride (Sigma-Aldrich). Nuclei were counterstained with hematoxylin (Vector Laboratories). For immunofluorescence, sections were stained with anti-Ki67 Alexa-488 (rabbit, D3B5, Cell Signaling Technology), followed by anti-CD3 (polyclonal rabbit, DakoCytomation) with a secondary biotin-labeled goat-anti-rabbit Ab and Streptavidin-DyLight 594 (Vector Laboratories). The sections were mounted with medium for fluorescence containing 4,6-diamidino-2-phenylindole (DAPI, Vector). Images were acquired using a Leica DM5500B upright microscope and LAS image acquisition software (Leica Microsystems, Rijswijk, The Netherlands).

### Statistical analysis

Significance was determined using Student’s *t*-test or one-way performed on GraphPad Prism 5.0 software (GraphPad Software, La Jolla, CA, USA), as indicated in the figure legends. *P* values of <0.05 were regarded as significant. In case of multiple comparisons, an adjusted significance level was used according to the Bonferroni correction (significance level = 0.05/number of comparisons).

## Supplementary Information

SUPPLEMENTARY FIGURES
