# Supplementary material for: Duplication of the IL2RA locus causes excessive IL-2 signaling and may predispose to very early onset colitis
Source: Mucosal Immunol. 2021 Jul 5;14(5):1172–82. doi: 10.1038/s41385-021-00423-5 (PMC8379074; doi:10.1038/s41385-021-00423-5)
Supplement: Supplementary file 1 — SUPPLEMENTARY FIGURES [file 41385_2021_423_MOESM1_ESM.pdf]

SUPPLEMENTARY FIGURES

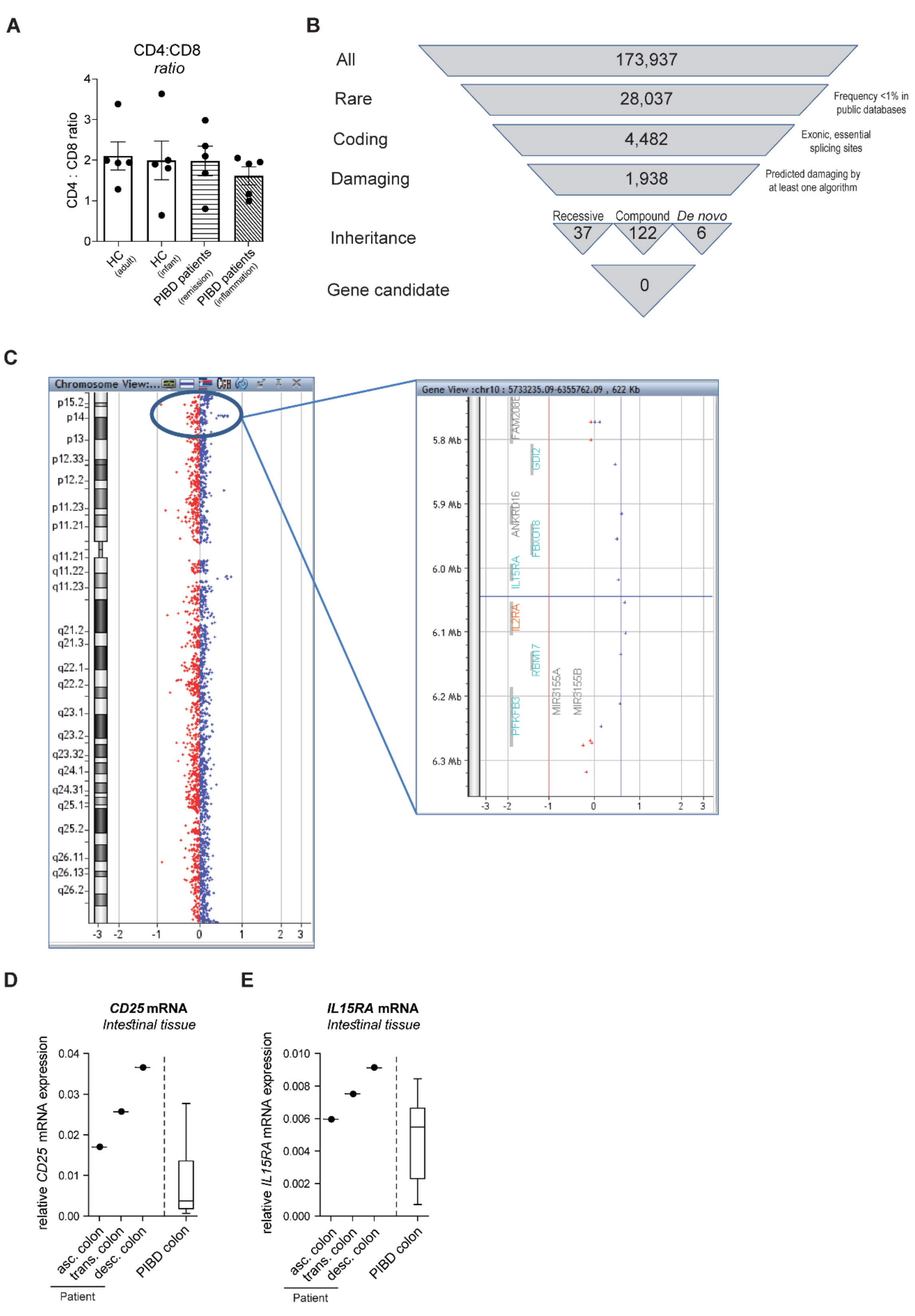

Figure S1. Identification of a 374 kb duplication on 10p15.1 including the IL2RA locus. (A) Flow cytometric analysis of CD4:CD8 ratio in peripheral blood of adult healthy controls (HC, adult n=5) and infant healthy controls (HC, infant age 1.3-3.4 years, n=5), PIBD patients with active intestinal inflammation (n=5) and PIBD patients in remission (n=5). (B) Whole exome sequencing analysis revealed >170,000 single nucleotide variants (SNVs). Filtering of SNVs is shown. First, rare (with a frequency of less than 1% in public databases) and coding variations (affecting exons or essential splicing site) that were predicted to be damaging for the protein function by at least one algorithm were selected. Then, inheritance disease modes were applied and candidate genes were screened regarding protein function. All in all, no disease-causing mutations were identified. (C) Cytogenetic analysis performed on peripheral lymphocytes. Array-CGH profile showing the 10p15.1 duplication (log2 ratio=+0.5). (D) Relative *CD25* mRNA and (E) *IL15RA* mRNA expression in total resected colonic tissue of the patient and PIBD patients (n=8).

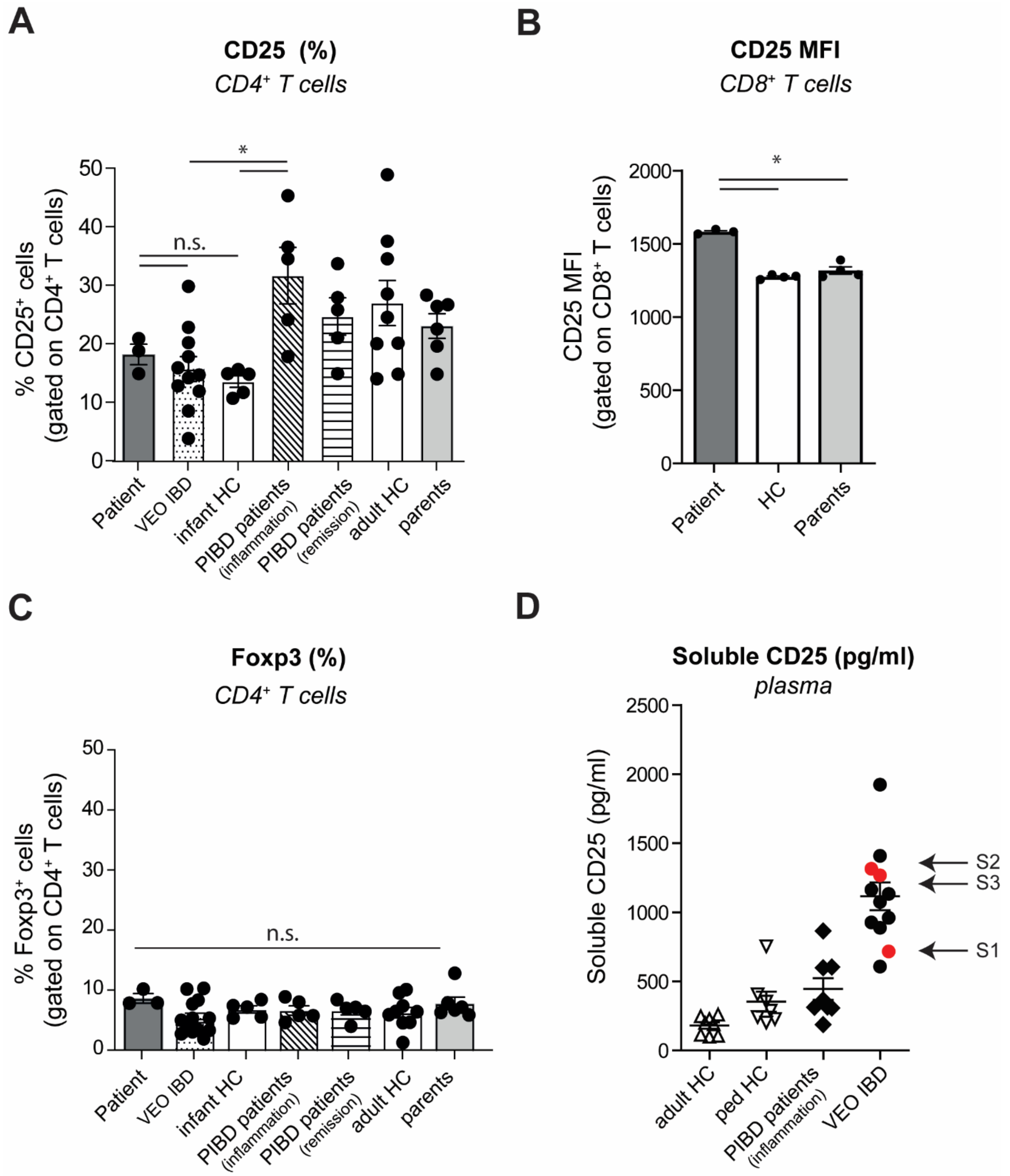

Figure S2. Frequencies of CD25⁺CD4⁺ T cells, Foxp3⁺CD4⁺ T cells and soluble CD25 concentrations in patient and controls. (A-C) Flow cytometric analysis of CD3, CD4, CD8, CD62L, CD38, CD25 and/or Foxp3 expression was performed on peripheral blood from adult healthy controls. (A) Frequencies of $CD25^+$ cells gated on total circulating $CD4^+$ T cells of the patient and her parents (time points S3 and S4); VEO-IBD patients (n=11); infant healthy controls (HC infant, age 1.3-3.4 years, n=5), PIBD patients with active intestinal inflammation (n=5) and PIBD patients in remission (n=5) and adult healthy controls (HC, adult n=9). (B) CD25 expression (MFI) on total circulating $CD8^+$ T cells (HC, n=4), the patient and her parents (time points S3 and S4). (C) Frequencies of regulatory $CD4^+Foxp3^+$ T cells in peripheral blood of the patient and her parents (time points S3 and S4); VEO-IBD patients (n=11); infant healthy controls (HC infant, age 1.3-3.4 years, n=5), PIBD patients with active intestinal inflammation (n=5) and PIBD patients in remission (n=5) and adult healthy controls (HC, adult n=9). (D) Plasma concentrations of soluble CD25 in adult healthy controls, pediatric healthy controls, PIBD patients with intestinal inflammation (n=8) and VEO-IBD patients (n=9). N.s., not significant, *$p<0.05$, **$p<0.01$, ***$p<0.001$ using one-way ANOVA followed by the Bonferroni's Multiple Comparison Test. PIBD, pediatric-onset IBD.

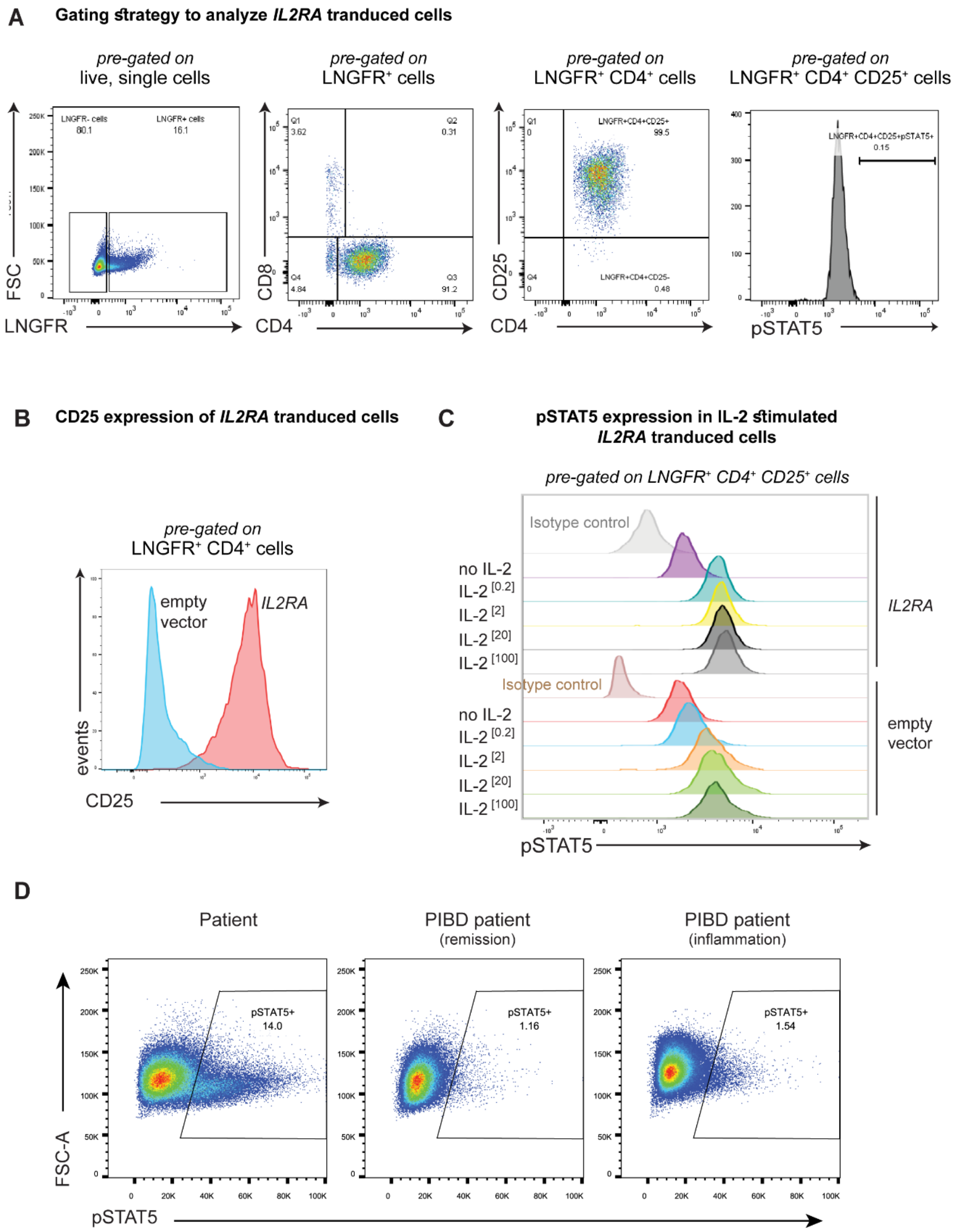

Figure S3. *In vitro* overexpression of CD25 increases responsiveness to IL-2 in CD4⁺ T cells. The *IL2RA* cDNA was retrovirally transduced in human healthy donor T cell blasts. Empty vector was used as a control. Cells were IL2-starved during 36h, and stimulated with increasing dosages of IL-2 (0.2 IU/ml to 100 IU/ml) for 20 minutes. IL-2-induced STAT5 phosphorylation (pSTAT5) in CD4⁺ CD25⁺ LNGFR⁺ T cells was then assessed by flow cytometry. (A) flow cytometric gating strategy. (B) CD25 expression on *IL2RA* or empty vector control transduced T cells (C) pSTAT5 expression in CD4⁺ CD25⁺ LNGFR⁺ T cells after IL-2 stimulation (representative experiment). (D) PBMCs of the patient, PIBD patients with active intestinal inflammation (n=3) and PIBD patients in remission (n=3) were stimulated with IL-2 (100 IU/mL) for 15 min followed by quantification of STAT5 phosphorylation (pY694) in CD4+ cells by flow cytometry (visit S3) Representative dotplots.

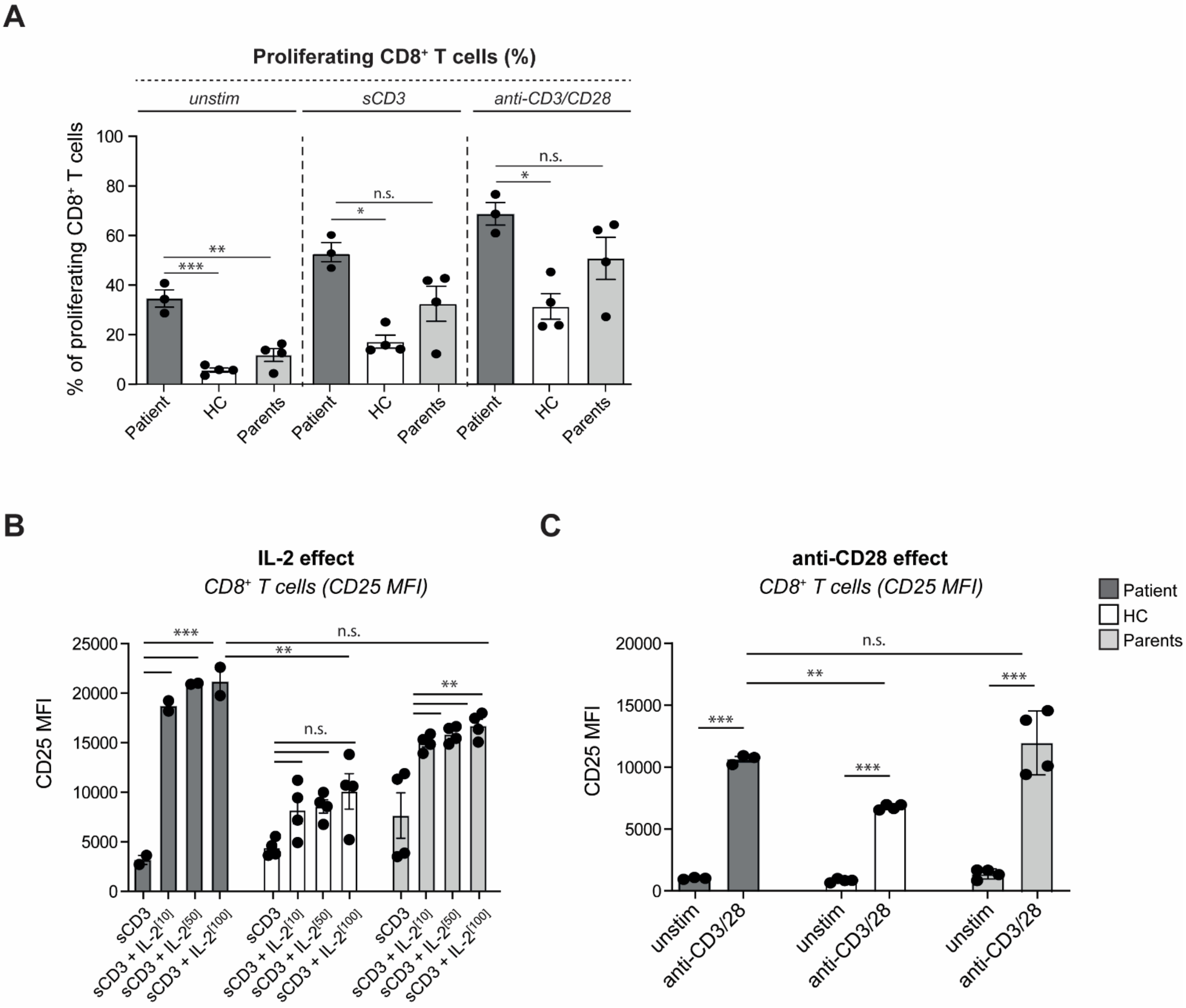

Figure S4. Frequencies of proliferating CD8$^+$ T cells and CD25 expression on CD8$^+$ T cells after TCR-ligation in combination with increasing doses of exogenous IL-2. (A) Healthy adult control and patient PBMCs (visits S3 and S4) were stimulated with anti-CD3 (500 ng/ml) or antiCD3/anti-CD28 beads (bead-to-cell ratio 1:2) for 48h. (A) Percentage of proliferating CD8$^+$ T cells was analyzed by CellTrace Violet dilution. (B) PBMCs were stimulated with anti-CD3 (500 ng/ml) in the absence or presence of IL-2 (1, 50 or 100 IU/ml). After 48h, cells were stained for CD3, CD8 and CD25 and analyzed by flow cytometry. (C) PBMCs were stimulated with anti-CD3/CD28 beads (bead-to-cell ratio 1:2). CD25 expression on CD8$^+$ T cells was analyzed at 48h. Data are mean ± SEM (n=4). Representative of two independent experiments (time points S3 and S4); N.s., not significant, *p<0.05, **p<0.01, ***p<0.001 using one-way ANOVA followed by the Bonferroni's Multiple Comparison Test.

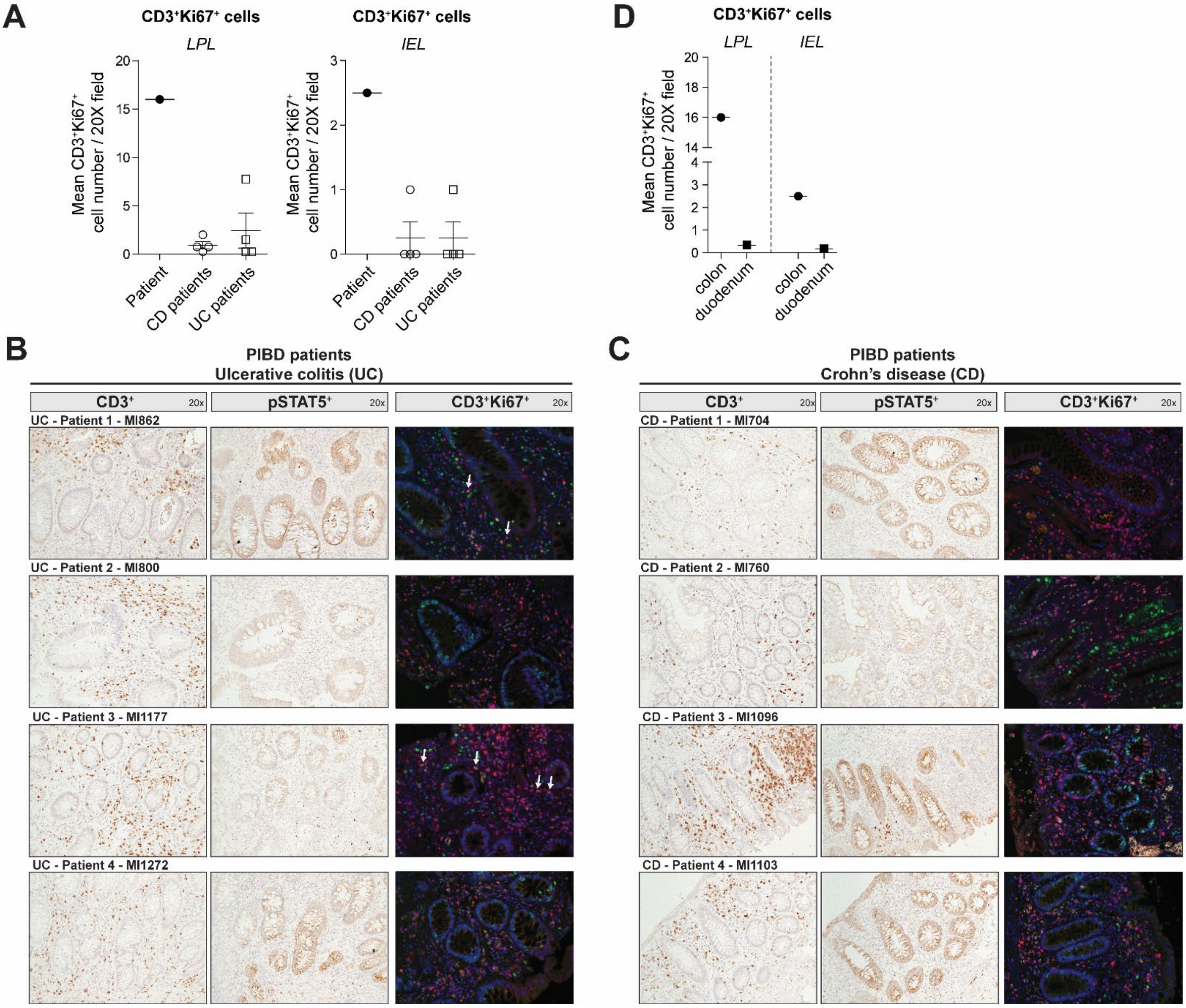

Figure S5. Low frequency of proliferating CD3⁺Ki67⁺ cell infiltration and low STAT5 phosphorylation in resected colonic tissue sections of PIBD patients. (A) Numbers of $CD3^{+}Ki67^{+}$ cells in resected colonic tissues of the patient and control PIBD patients were quantified in blinded fashion by counting four 20X images. (B-C) Representative immunohistochemical staining for CD3 and pSTAT5 and immune fluorescence for CD3 and Ki67 in paraffin-embedded resected inflamed colonic tissue of treatment-resistant pediatric-onset UC patients (n=4) and treatment-resistant pediatric-onset CD patients (n=4). Green=Ki67, red=CD3, blue=4',6-diamidino-2-phenylindole (DAPI) nuclear staining. (D) Numbers of $CD3^{+}Ki67^{+}$ cells in the patient's colonic inflamed biopsies were compared to non-inflamed duodenal tissue obtained during initial assessment at diagnosis.

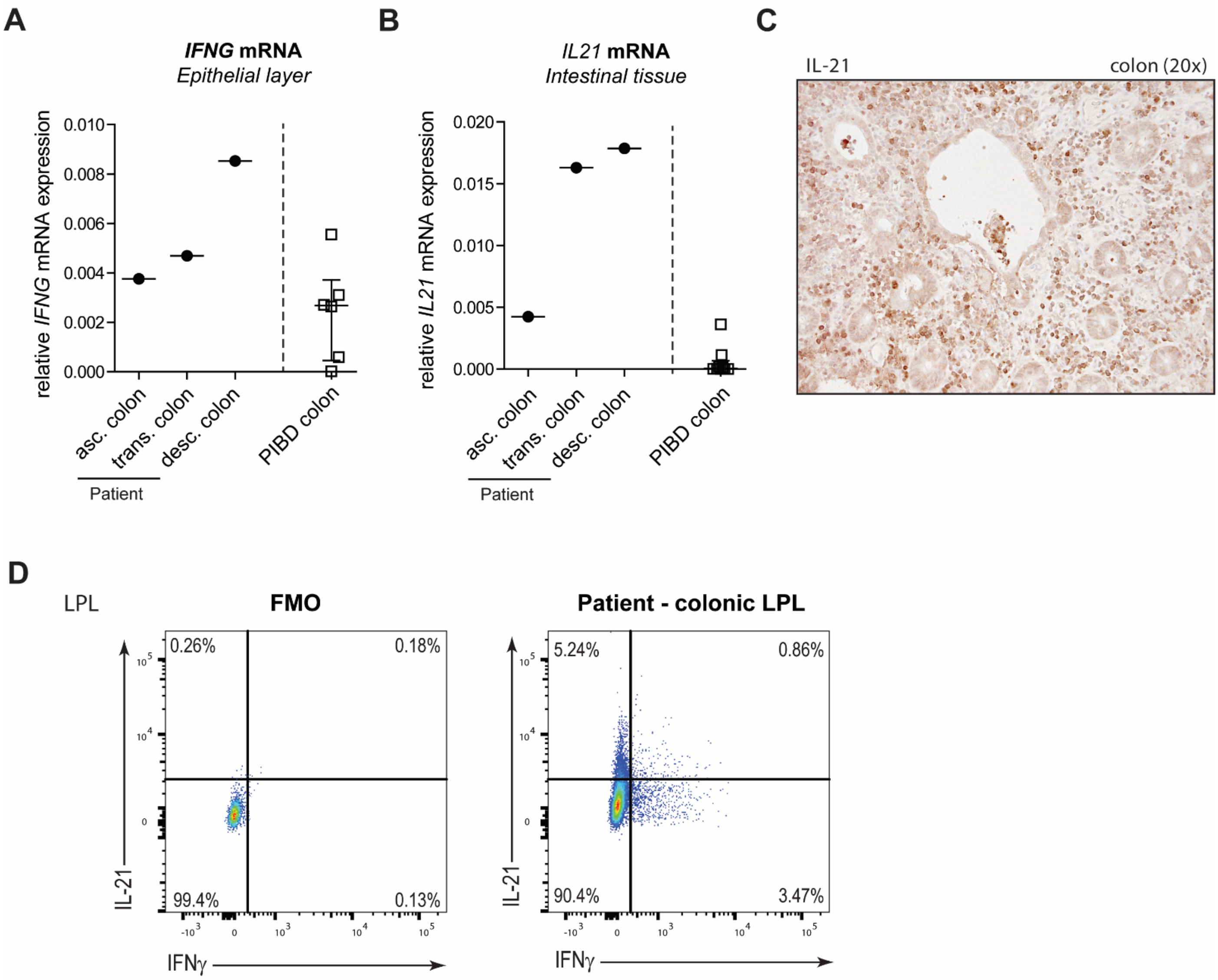

Figure S6. IL-21⁺ and IFNγ⁺ cells in inflamed patient colonic tissue. (A) *IFNG* mRNA expression in epithelial layers isolated form resected colonic tissue in the patient and PIBD patients. (B) *IL21* mRNA expression in total resected colonic tissue of the patient and PIBD patients. (C) Representative immunohistochemical staining for IL-21 in paraffin-embedded resected inflamed colonic tissue (visit S2). (D) LPL were isolated from patient inflamed colonic tissue and stimulated with PMA (0.02 µg/ml) and ionomycin (0.5 µg/ml). Frequencies of IL-21⁺ and IFNγ⁺ cell in CD3⁺ LPL were analyzed by flow cytometry.
